# Supplementary material for: Relations of Bedtime Mobile Phone Use to Cognitive Functioning, Academic Performance, and Sleep Quality in Undergraduate Students
Source: Int J Environ Res Public Health. 2020 Sep 29;17(19):7131. doi: 10.3390/ijerph17197131 (PMC7579316; doi:10.3390/ijerph17197131)
Supplement: Supplementary file 1 [file ijerph-17-07131-s001.pdf]

Supplementary Materials –The Bedtime Mobile Phone Use Scale

Each item refers to behaviors that have occurred at night after lights out in the last 30 days. Select the best answer that describe you.

Never = 1, One to three times a month = 2, Once a week = 3, Several times a week = 4, Every day = 5

No.	Items	Never	One to three times a month	Once a week	Several times a week	Every day
I.	How frequently do you receive text messages after lights out?	1	2	3	4	5
II.	How frequently do you read text messages received after lights out?	1	2	3	4	5
III.	How frequently do you send text messages after lights out?	1	2	3	4	5
IV.	How frequently do you receive phone calls after lights out?	1	2	3	4	5
V.	How frequently do you answer phone calls received after lights out?	1	2	3	4	5
VI.	How frequently do you make phone calls after lights out?	1	2	3	4	5
